# Molecular docking analysis of quercetin with known CoVid-19 targets

**DOI:** 10.6026/973206300191081

**Published:** 2023-11-30

**Authors:** Sridevi Chandra Subash Chandran, Ireen Christopher, Aishwariya Sounderraajan, Viji Murugesan, Indu Sabapathy, Vijayalakshmi Periyasamy, Rajalakshmi Manikkam

**Affiliations:** 1 Sastra Deemed University, Tirumalaisamudram, Thanjavur - 613401, India; 2 Department of Biotechnology and Bioinformatics, Holy Cross College (Autonomous), Tiruchirappalli, Tamil Nadu, India; 3 DBT-BIF Centre, Holy Cross College (Autonomous), Tiruchirappalli, Tamil Nadu, India; 4 Department of Zoology, Holy Cross College (Autonomous), Tiruchirappalli, Tamil Nadu

**Keywords:** CoVid-19, quercetin, remdesivir, ADMET, docking

## Abstract

Combat and care during CoVid-19 was non-trivial. Therefore, it is of interest to use the pharmacologically active plant component quercetin for the treatment of CoVid-19. Quercetin exhibits favourable ADMET values and abides by Lipinski's rule of five. When quercetin and remdesivir were positioned in relation to the CoVid-19 targets, quercetin exhibited a greater propensity for binding and H-bond interaction in their molecular interactions. Thus, the quercetin molecule can be used to manage CoVid-19.

## Background:

Many of the epidemic and pandemic outbreaks of zoonotic infections like swine flu (H1N1), Bird flu (H5N1), Ebola, MERS, SARS and recent pandemic outbreak CoVid-19 made the scientific and research community baffled about the origin, spread and treatment strategies [1]. Respiratory disease in human (CoVid-19) is caused by coronavirus also known as SARS-COV-2. The enveloped, non-segmented, single-stranded RNA virus known as SARSCoV-2, which belongs to the Sarbecovirus subgenus of the Coronaviridae family, has four essential proteins on its surface: spike S glycoprotein, envelope protein (E), membrane protein (M), and nucleocapsid protein (N). These proteins define the ultrastructure of the SARS-CoV-2 virus. This disease causes mild to severe respiratory symptoms which can result in lung damage or death in number of cases [2]. Angiotensin-converting enzyme 2 (ACE2) and transmembrane protease serine 2 (TMPRSS2) are required for SARS-CoV-2 to enter its host cell. SARS-CoV-2 infection is more likely to occur in human cells with abundant ACE2, a crucial modulator involved in controlling blood pressure. The structure of the SARS-crystal CoV-2 showed that its receptor-binding domain (RBD) has a higher propensity to bind to the human ACE2, according to recently obtained data. For the virus to enter cells, its protein S must attach to the ACE2 receptor with the aid of certain host cell proteases like TMPRSS2. [3,4]. Since the beginning of the pandemic, remdesivir [5], molnupiravir [6], favipiravir [7], and monoclonal antibodies like bamlanivimab [8] have all been reported to be effective against CoVid-19. Pfizer, Moderna, Johnson & Johnson, and Astra Zeneca developed and marketed several immunisations to prevent CoVid-19 [9]. The process of creating new medications for CoVid-19 has grown arduous because of the pandemic condition. In that situation, an *in-silico* study opens the door for analysing the current medication so that it can be used for CoVid-19, such process is called drug Repurposing, one of the booming fields in the research area. Finding new remedies for ailments can be expedited and made less expensive with its help, especially if preclinical safety studies have already been completed [10]. It can play an important role in the "therapeutic stratification procedure" for patients with uncommon, difficult, or chronic diseases with few or no commercially available therapy alternatives [11]. CoVid 19 made use of several anti-inflammatories, antimalarial, and antiparasitic medications, as well as nucleoside analogues and monoclonal antibodies. Numerous herbal items, vitamins, minerals, and immune system boosters were advertised as alternatives to medications and vaccines for the prevention and treatment of CoVid-19 [12]. Onions, grapes, berries, cherries, broccoli, and citrus fruits are the main sources of quercetin. It is a yellowish crystal that is soluble in acetic acid/alkali solutions but insoluble in water [13]. Alcohol has a weak solubility. Due to the upregulation of pro-inflammatory molecules like inflammasomes, cytokines, and interleukin 1β and 18 in CoVid-19, the second phase is associated with a cytokine storm [14]. As a result, immune stimulants and anti-inflammatory drugs are suggested throughout the first and second phases. Quercetin, a well-known antioxidant, has anti-inflammatory and immune-modulating effects. Quercetin is therefore thought to have all the qualities needed for CoVid-19 therapy [15]. Remdesivir is a nucleoside analogue that is used to treat infections caused by RNA viruses, such as CoVid-19. Remdesivir has garnered a lot of interest as a potential CoVid-19 treatment [16] since it was initially shown to have efficacy against the Coronaviridae family in 2017 [17]. Therefore, it is of interest to document the molecular docking analysis of quercetin with CoVid-19 targets

##  Materials and methods:

## Target preparation:

The CoVid targets WNT (6AHY_B), FZD (6AHY_A), Lrp-6(4A0P_A), spike protein (6VSB_C), ACE2(7U0N_A), and TMPRSS2(6DK5_A) were downloaded from PDB database in PDB format. The obtained targets were prepared in Discovery Studio Biovia visualiser client 2021 by removing the water molecule, nucleic acid groups, native ligand groups, HETATM and adding polar hydrogen bond.

## Ligand preparation:

**Figure 1:**
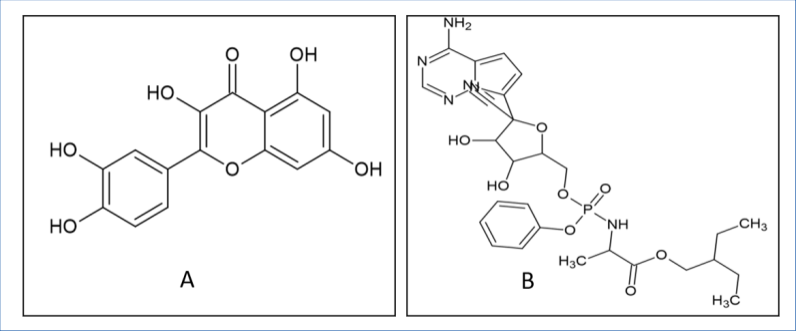
2D structures of quercetin and remdesivir derived from Chemsketch

The structures of the ligand were downloaded from PubChem database in SDF format and 3D structure of the ligands (drugs) were generated using ACD/labs Chemskectch. Remdesivir is used as a reference drug in this study (Figure 1).

## Prediction of drug likeliness properties:

The Lipinski rule of five and ADMET parameters were calculated using the PKCSM website to explain the selected ligands' drug-like characteristics.

## Docking:

**Figure 2:**
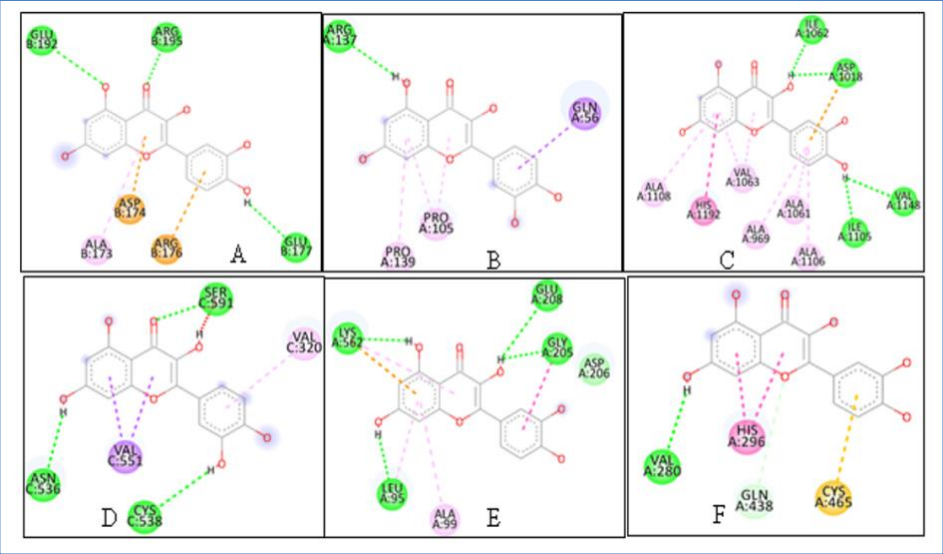
Molecular interaction of Quercetin with the CoVid-19 targets (A) WNT (B) FZD (C) LRP-6 (D) spike protein (E) ACE2 and (F) TMPRSS2

**Figure 3:**
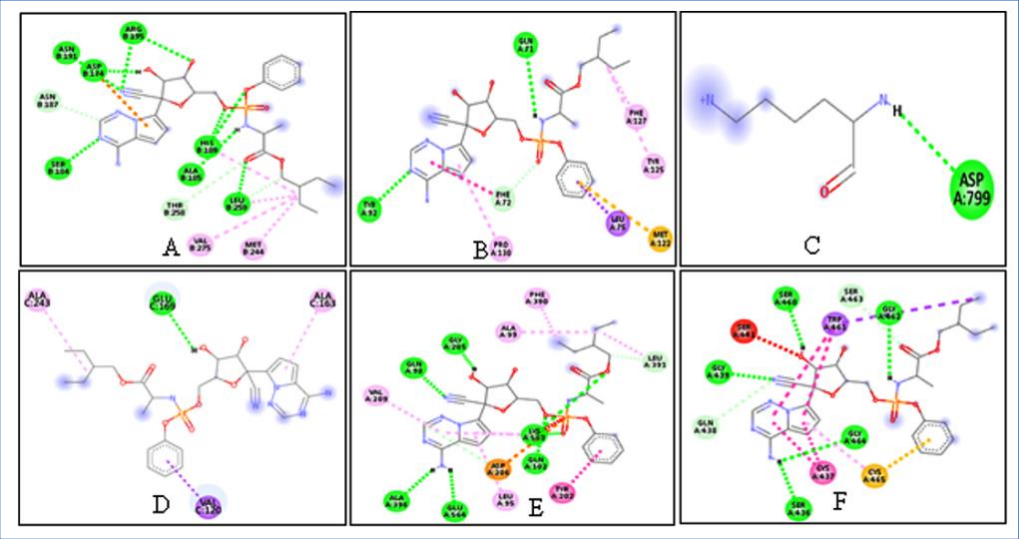
Molecular interaction of remdesivir with the CoVid-19 targets (A) WNT (B) FZD (C) LRP-6 (D) spike protein (E) ACE2 and (F) TMPRSS2

The Lamarckian genetic approach was used to do the molecular docking analysis utilising the PyRx-v0.8 virtual screening tool in conjunction with Auto Dock-Vina. Initially, the ligand was standardised and put into PDBQT format. The ligands were inserted through open babel and the Universal Force Field (UFF) served as the energy reduction parameter and the optimisation algorithm used conjugate gradient descent. The chosen target proteins were independently docked with the ligands (Quercetin (Figure 2) and remdesivir (Figure 3)) in separate docking runs. The ligand was assumed to be flexible and the protein to be rigid throughout the docking step. The grid configuration file was created using the Pyrex Auto Grid engine. The implementation was also utilised to determine or anticipate which amino acids would interact with ligands at the location of the active protein. To forecast the binding modes and docking energies, blind docking technique were used. RMSD (root-mean-quarter deviation) values less than 1.0 were regarded as ideal. In order to come up with a good binding, they are gathered together. A ligand with a high binding affinity was discovered to have the highest binding energy (most negative).

## Results and Discussion:

The demand for innovative, more adaptable diagnostic techniques for the detection of SARS-CoV-2 infections has been fuelled by the advent of novel and changing SARS-CoV-2 variants. On the other hand, it is currently more difficult to develop quick and accurate diagnostic tools due to novel variations and a variety of symptoms presented by infected individuals. Additionally, vaccinations remain the cornerstone of infection defence and prevention. To boost immunity and effectively combat SARS-CoV-2 and its variants, new drugs and vaccines are constantly being developed. Drug repurposing is one of the techniques to develop the drug for CoVid 19. Numerous existing drugs and plant based phytocompounds are being repurposed for CoVid 19, one of the plant-based components is quercetin which possesses number of pharmacological properties. Supplementing with quercetin has been shown to have antioxidant [18], anti-inflammatory, immunoprotected, anticarcinogenic, and antidiabetic effects, as well as the capacity to inhibit lipid peroxidation, platelet aggregation, capillary permeability, and to stimulate mitochondrial biogenesis [19,20, 21]. Quercetin is being employed more frequently in fresh formulations for human health care due to its high solubility and bioavailability [22]. Due to its potential antiviral actions in inhibiting polymerases, reverse transcriptase, and proteases, reducing DNA gyrase, and binding viral capsid proteins, quercetin has also been examined in many types and models of viral infection [23, 24, 25, 26]. In CoVid-19 symptomatic patients, quercetin was recently employed as adjuvant therapy; the outcome was an improvement in clinical symptoms and a shortening of the hospital stay [27]. Clinical trials using zinc, vitamin C, curcumin, vitamin D3, masitinib, hydroxychloroquine, azithromycin, and ivermectin in combination with quercetin-based monotherapy and combination therapy against CoVid-19 were encouraging [28].

The previous study found that quercetin has a bioavailability of 24-53% from the stomach, has no negative effects on the CYP450 enzymes, and does not deplete the glutathione enzyme. It also found that quercetin is metabolized to two non-carcinogenic compounds, 3'-O-methyl quercetin, and 4'-O-methyl quercetin, and is eliminated through the urine and feces (Grass Notices). Due to the pandemic situation, the process of developing drugs for CoVid 19 has become a tedious process. In that case, an *in-silico* study paves the way to analyze the existing drug in order to use it for CoVid-19. This work is to examine the action of quercetin against CoVid 19 targets. The drug likeliness property of the drug is important, Quercetin obeys Lipinski rule of five (Table 1), which states that the quercetin follows the rules with just one violation with seven acceptors. In ADMET (Table 2), quercetin has 77% of intestinal absorption were as remdesivir has 71%, it does cause side effects in brain because the value for BBB and CNS are equal to <-1 and <-3 respectively.

Quercetin is not a substrate or inhibitor for CYP2D6 and CYP3A4. The compound is a not an inhibitor for hERG which does not cause QT prolongation in heart and the hepatotoxicity of the compound is negative, it doesn't lead to liver injury, but the reference drug shows the hepatotoxicity. To know the potential of quercetin against CoVid 19 targets, molecular docking was done (Table 3).

Quercetin (Figure 2) was interacted with six targets (Wnt -3, Frizzel, Lrp 6 A chain, Spike protein, ACE2, TMPRSS2) among them quercetin shows higher binding affinity towards LRP, WNT-3 and ACE-2 as (-9.3, -8.3 and -8.3) respectively. For LRP 6 the compound shows four hydrogen bond interactions (ILE-1062, ASP-1018, VAL-114, ILE-1105) for WNT-3 it shows three hydrogen bond interactions (GLU-96, ARG-195, GLU-177) and for ACE-2 it shows five hydrogen bond interactions (LYS-562, GLU-208, GYL-205, ASP-206, LEU-95). Thus, this study's findings suggest that quercetin can be utilised to manage CoVid 19.

## Conclusion:

The plant-based compound quercetin obeys drug likeliness properties and it shows better binding affinity and H-bond interaction to targets associated with CoVid-19 for further consideration.

## Figures and Tables

**Table 1:** Comparison of the drugability of Quercetin and Remdesivir

**Compound name**	**Molecular Weight**	**LogP**	**Rotatable Bonds**	**Acceptors**	**Donors**	**Surface Area**
Quercetin	302.238	1.988	1	7	5	122.108
Remdesivir	602.585	2.31218	13	13	4	242.488

**Table 2:** ADMET properties of Quercetin and Remdesivir

**Property**	**Model Name**	**Quercetin**	**Remdesivir**
	Water solubility	-2.925	-3.07
	Caco2 permeability	-0.0229	0.635
Absorption	Intestinal absorption (human)	77.2	71.109
	P-glycoprotein substrate	YES	YES
	VDss (human)	1.559	0.307
	Fraction unbound (human)	0.206	0.005
Distribution	BBB permeability	-1.098	-2.056
	CNS permeability	-3.065	-4.675
Metabolism	CYP2D6 substrate	NO	NO
	CYP3A4 substrate	NO	YES
	CYP2D6 inhibitior	NO	NO
	CYP3A4 inhibitior	NO	NO
Excretion	Total Clearance	-0.407	0.198
	Renal OCT2 substrate	NO	NO
	hERG I inhibitor	NO	NO
	hERG II inhibitor	NO	YES
	Oral Rat Acute Toxicity (LD50)	2.47	2.043
	Hepatotoxicity	NO	YES

**Table 3:** Binding affinity and Hydrogen bond interaction of Quercetin and Remdesivir with the targets of interest

**Targets**	**Binding affinity**		**H-bond interactions**		
	**Quercetin**	**Remdesivir**	**Quercetin**	**Remdesivir**
Wnt -3	-8.3	-8	GLU-96, ARG-195, GLU-177	ASN B-191, ASP-174, ARG-195, SER-184, HIS-189, ALA- 185, LEU-259
Frizzel	-6.6	-6.4	ARG-137	GLY-71, TYR-92
Lrp 6	-9.3	-8.4	ILE-1062, ASP-1018, VAL-114, ILE-1105	ASP -799
Spike protein	-6.9	-6.1	SER-591, ASN-536, CYS- 538	GLU-169
ACE2	-8.3	-7.9	LYS-562, GLU-208, GYL-205, ASP-206, LEU-95	GLY A:205, GLN A:98, LYS A:562, GLN A:102, ALA A:396,GLU A:564
TMPRSS2	-7.3	-7.3	VAL-280	SER A:460, GLY A:439, GLY A:462, GLY A:464, SER A:436
